# The Role of Virtual Reality, Exergames, and Digital Technologies in Knee Osteoarthritis Rehabilitation Before or After Total Knee Arthroplasty: A Systematic Review of the Interventions in Elderly Patients

**DOI:** 10.3390/medicina61091587

**Published:** 2025-09-02

**Authors:** Ludovica Di Curzio, Teresa Paolucci, Sandra Miccinilli, Marco Bravi, Fabio Santacaterina, Lucrezia Giorgi, Silvia Sterzi, Loredana Zollo, Andrea Bernetti, Federica Bressi

**Affiliations:** 1 Università Campus Bio-Medico di Roma, 00128 Rome, Italy; 2 Università degli Studi di “G. D’Annunzio”, 66100 Chieti-Pescara, Italy; 3 Fondazione Policlinico Universitario Campus Bio-Medico, 00128 Rome, Italy; 4 Università del Salento, 73100 Lecce, Italy

**Keywords:** knee osteoarthritis, rehabilitation, technologies, virtual reality, exergames, telerehabilitation, compliance, pain, stability, functionality

## Abstract

*Background and Objectives*: Osteoarthritis (OA) is a chronic, degenerative joint disease. The main symptoms include pain that can cause loss of function and stiffness, as well as swelling, reduced range of motion, crepitus, joint deformity, and muscle weakness. It leads to irreversible structural changes, that in advanced stages can require surgical interventions. The aim of this review was to summarize the current literature about the role of virtual reality (VR), exergames and digital technologies in patients with knee osteoarthritis before or after total knee arthroplasty, to understand if it is possible to prevent and reduce the symptoms and if these new technologies are more effective than conventional rehabilitation therapies. *Materials and Methods*: We conducted a systematic search of PubMed, Cochrane Library, Scopus, and PEDro from inception to November 2024. The review adhered to the PRISMA 2020 guidelines, and the protocol was prospectively registered in PROSPERO (registration number: CRD42024541890). We included randomized controlled trials (RCTs) enrolling participants aged 60 years or older, in which VR or telerehabilitation programs were compared with conventional rehabilitation approaches. Eligible studies had to report at least one of the following outcomes: pain, functionality, stability, or adherence. Two independent reviewers screened titles and abstracts, assessed full-text eligibility, extracted data, and evaluated the risk of bias using the Cochrane Risk of Bias 2 (RoB 2) tool. *Results*: Fourteen randomized controlled trails (RCTs) (1123 participants; mean age 68.2 years) were included. VR and telerehabilitation generally outperformed conventional rehabilitation for pain (8/13 studies, −0.9 to −2.3 VAS points) and functionality (7/13 studies, WOMAC improvement 8–15%, TUG −1.2 to −2.8 s). Compliance was higher in most technology-assisted programs (6/7 studies, 70–100% adherence). Stability outcomes were less consistent, with only 1/4 studies showing clear benefit. One study favored conventional rehabilitation for functionality. Overall risk of bias was low-to-moderate, with heterogeneity mainly driven by intervention duration, platform type, and supervision level. *Conclusions*: Structured telerehabilitation, non-immersive VR, and interactive online exercise programs, especially those offering real-time feedback, show comparable or superior benefits to conventional rehabilitation in older adults with knee OA or after TKA, particularly for pain reduction, functional gains, and adherence. These approaches enhance accessibility and home-based care, supporting their integration into clinical practice when in-person therapy is limited.

## 1. Introduction

OA is a chronic condition that affects the joints and their associated tissues. It primarily leads to a gradual deterioration of the articular cartilage, which eventually impacts the subchondral bone and the adjacent synovial structures [1].

Joints maintain a constant dynamic equilibrium between the continuous formation and breakdown of the cartilaginous matrix. This balance is regulated by the interplay between anabolic and catabolic pathways, which alter the metabolic activity of chondrocytes. When harmful pathways exceed the body’s compensatory capacity, and an impairment in chondrocyte function occurs, degradation of the cartilaginous matrix ensues [2].

According to the World Health Organization, 2019 updates show that osteoarthritis affects approximately 9.6% of men and 18.0% of women over the age of 60. In the coming years, the global incidence of osteoarthritis is set to rise significantly, and this means that the healthcare system must recognize that it is potentially becoming one of the most common diseases worldwide [3]. Preventive target treatments may help restore the altered chondrocyte phenotype and prevent further progression of the disease [4]. Globally it is considered as a major public health concern due to the economic burden it causes [5]. Knee involvement represents the greatest disability concern from a public health perspective, as it causes pain and loss of function in an estimated 10% to 15% of men and women over the age of 45 [6]. Due to the combined effects of an aging population, rising obesity rates, and a growing number of joint injuries, OA is becoming increasingly prevalent [3].

Increasing symptoms and knee pain can lead to a significant and persistent decline in everyday activities [7]. Treatments such as physiotherapy, orthopedic aids, medications, and surgery try to relieve the symptoms and the clinical manifestations that occur. Primary prevention of osteoarthritis of the knee could be the most effective approach [2]. Pharmacological treatment options are still limited, and non-pharmacological treatments are often underutilized [8]. It has a big impact on the quality of life because it is also combined with physiological frailty and multiple coexisting medical conditions [9,10].

In the last ten years, there has been a significant increase in research, exploring the use of virtual reality and gaming technologies within the elderly population. The incorporation of exercise through video games is increasingly utilized to enhance physical activity, improve overall health, and maintain physical function in older adults. They can be used in home environments and in outpatient and hospital facilities. Thanks to them, even socio-economic expenditure can be reduced to the advantage of a better quality of life for all patients trying to reduce severe conditions and surgical interventions in order to give patients all the same possibility of treatment, near and far away from centers of excellence [11,12].

Moreover, these new technologies are being demonstrated to be useful since they can be tailored to various difficulty levels and adjusted in real-time during training. With technology becoming more affordable and accessible in recent years, its benefits are being recognized across diverse settings and conditions [11].

Therefore, the aim of this systematic review is to contribute with an updated analysis of the current literature on the existing evidence regarding the impact of new technologies (virtual reality, exergames and telerehabilitation) in older adults with knee OA, to understand the compliance and beneficial clinical effects of these new therapies. This review takes into consideration different outcomes such as compliance to therapy, pain, stability and functional recovery.

## 2. Materials and Methods

A thorough systematic review of the literature was carried out following the methodological guidelines outlined in the PRISMA 2020 statement [13]. The protocol was previously registered in the PROSPERO database under the registration number CRD42024541890.

### 2.1. Data Source and Study Searching

The literature research was initially carried out through an electronic search, evaluating the inclusion criteria we had established. Then a manual electronic search was conducted in November 2024, using search terms; no time restriction was applied and the research was restricted to English language publications found in the following databases: PubMed, Scopus, Cochrane Library and PEDro. Search terms with Boolean operators (Or/AND) were used. Keywords used were as follows: knee osteoarthritis, virtual reality, exergames, rehabilitation, technologies. Mesh Terms were used on PubMed, and on Scopus, TITLE-ABS-KEY was used.

### 2.2. Eligibility Criteria and PICO Principle

The inclusion criteria we decided to establish are the following: elderly patients (older than 60 years old) with knee osteoarthritis or post total knee arthroplasty (TKA) for knee osteoarthritis.

The PICO principle was used to create a well-defined research question [14].

Participants (P): elderly patients (older than 60) with knee osteoarthritis or post TKA (total knee arthroplasty) for knee osteoarthritis. Trials including younger participants were retained if the mean age was ≥60 years or if subgroup/secondary analyses for older adults were reported;

Intervention (I): virtual reality, exergames, and tele-knee rehabilitation;

Comparisons (C): conventional treatment (face to face), no treatment, blended treatment, treatment with a physiotherapist or healthy participants;

Outcome (O): patients’ compliance, pain, functionality and stability;

Timing of outcome measurement (T): before and after the intervention;

Settings (S): inpatient or outpatient settings;

Study design (SD): randomized controlled trial (RCT).

### 2.3. Data Extraction and Data Analysis

Two independent reviewers (L.D.C. and L.G.) separately screened the articles. All the papers were initially evaluated by title and abstract. Subsequently, the authors independently assessed the full-text versions of each publication and excluded those that did not meet the inclusion criteria. Data extraction from the included studies was made using a structured form, and two reviewers (L.D.C., L.G.) independently checked it. A qualitative synthesis analysis was performed considering the selected studies. The following data were extracted from each article: author and year of publication, study design, the population demographics and baseline characteristics, details on intervention and control conditions, technologies used in the experimental groups, outcomes measures and scales, time of measurements. Discrepancies were identified and resolved through discussion with a third reviewer (F.B.). Due to the heterogeneity of the data found, it was not possible to carry out a meta-analysis. An AI-Powered Systematic Review Management Platform (Rayan) was used for the systematic review management.

### 2.4. Risk of Bias Assessment

The risk of bias was assessed with the Cochrane RoB 2.0 tool [15], which evaluates the risk in five key areas. The risk was analyzed and assessed independently by two reviewers (L.G. and L.D.C). In case of inconsistent results, they were discussed with a third reviewer (F.B.). Any difference of opinion or result was resolved with a third party (F.B.).

### 2.5. Statistical Analysis and Summary of Findings

It was not possible to perform the intended statistical analysis and summary of findings as described in our protocol, due to heterogenic reporting style and lack of data in the individual studies included in this review. Thus, the effect on individual outcomes and overall quality assessment are narratively described. Only the data that were available in the studies were used. Authors of the studies included were not contacted for further information.

## 3. Results

**Figure 1:**
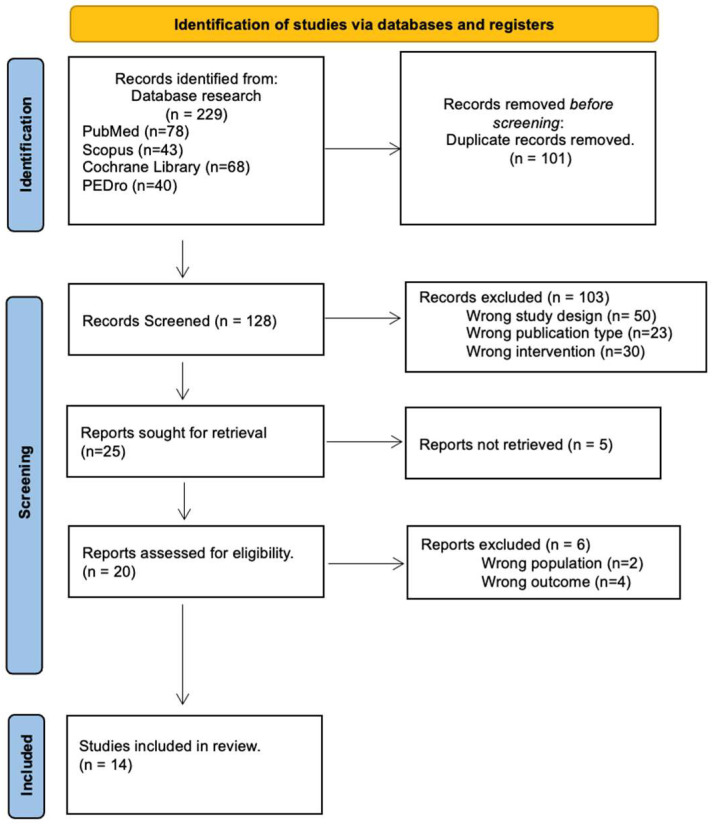
Flow chart of study selection according to PRISMA guidelines.

The search criteria returned 229 articles, and then 101 articles were removed because they were duplicates. Articles not RTC but reviews, systematic reviews and meta-analysis were excluded. A further 128 articles were screened, and 114 were excluded, resulting in 14 studies fulfilling the criteria for inclusion in this review. The detailed literature screening process can be found in Figure 1. A selection process was carried out. The studies considered and the outcomes reported are summarized in Table 1.

### 3.1. Compliance to the Therapy

Seven studies specifically evaluated whether the introduction of new technologies in the rehabilitation protocols could improve patient compliance with the therapy. Compliance to the therapy showed that six studies had a higher compliance and adherence to the treatment compared to conventional treatments. Only one study [25] reported that the telerehabilitation program had the same adherence to the traditional rehabilitation treatments. Compliance was evaluated through different questionnaires that were self-administered by the patients.

A recent study conducted by Moutzouri et al. [17] investigated the effectiveness of a 6-week web-based rehabilitation program combined with outdoor physical activity compared to an outdoor physical activity program alone. The findings showed that adherence to the web-based exercise component reached approximately 70%, in contrast to 48% in the control group, suggesting a positive impact of digital support on patient compliance.

Similarly, another study [18] evaluated the potential efficacy and acceptability of an eight-week, automated, internet-delivered pain-coping-skills training program known as Pain COACH. The attrition rate was minimal, with only 5% (*n* = 3) of participants withdrawing from the control group and 2% (*n* = 1) from the Pain COACH group. Specifically, in the Pain COACH group, adherence to the program was very high since 53 of the 58 (91%) participants completed all eight training modules.

In a study performed by Zhao et al. [24], patients in the telerehabilitation group exercised with the wearable device for an average of 98 min per week, achieving an overall training completion rate of 72%, and a training compliance rate of 90%. Furthermore, satisfaction with the application and wearable technology was reported at 94%, indicating a high level of acceptance and usability among users.

The study conducted by Doiron-Cadrin et al. [25] showed that all participants (100%) enrolled in the telerehabilitation group felt they had successfully achieved their prehabilitation goals and expressed overall satisfaction with the physiotherapy services received. Furthermore, 91% of participants considered the telerehabilitation program to be as effective as traditional rehabilitation. When using the Reacts liteVR app, only 36% of users reported that the sound and image quality remained stable throughout their telerehabilitation sessions, and the same proportion found the app to be user-friendly. Despite these technical challenges, related to software performance and internet connectivity, overall satisfaction with the telerehabilitation experience remained high, underscoring its potential feasibility and acceptability as an alternative to traditional rehabilitation delivery models.

The study conducted by Ga Yang Shim et al. [27] showed a higher number of system logins than exercise completions were observed in the DR group, likely due to technical issues. Some participants reported that UINCARE Home + system was difficult to use, citing software errors, challenges with usability, and installation inconveniences. In fact, the installation inconveniences were one of the reasons for several dropouts in the DR group. On the other hand, despite these issues, a satisfaction questionnaire completed by 11 patients showed a mean score > 3 for all questions, indicating a high level of satisfaction with the digital healthcare system and services.

Another study conducted by Trevor G Russell et al. [28] showed that, overall, patients were very satisfied with the service. Many patients reported that they would choose this rehabilitation method again and would recommend it to family and friends.

Lastly, in a study performed by Bettger et al. [26], the findings demonstrated that the VPT patients had higher adherence to the prescribed exercises, with patients engaging in the therapy on an average of 5.9 days per week, compared to 3.3 days per week in the conventional PT control group.

### 3.2. Pain

Analyzing the thirteen studies that evaluated the improvement in the pain perception when performing the rehabilitation treatment with the aid of technologies, we found that four studies used the VAS scale, four studies used the NRS scale, three studies used the WOMAC pain subscale and two studies used questionnaires. We can affirm that eight studies are in favor and show a significant reduction in pain, whilst five studies [25,26,27,28,29] show a neutral point of view, showing no significant reduction between groups. Different assessment tools were used to evaluate pain and different groups were analyzed without considering the different pain baselines.

Regarding the VAS scale, the results showed that VAS scores were significantly lower in the experimental group compared to the control group (*p* < 0.05) [23], with results going from 7.39 ± 1.14 on day one to 3.87 ± 0.55 on day seven in the experimental group. Pain scores, measured by the VAS, decreased in both groups. The reduction in pain in both groups was significant with a large effect size (*p* < 0.001) [19], with results going from 7.45 ± 1.19 to 2.70 ± 1.97 with a mean difference 4.75 (3.45 to 6.05). The last study [28] showed that telerehabilitation produced results comparable to those of conventional rehabilitation in terms of pain reduction, as measured by the VAS scale. The study [29] showed that there were no significant differences in VAS pain scores between the interactive virtual telerehabilitation group and the conventional therapy group, either at the end of the 2-week rehabilitation program or at the 3-month follow-up. This suggests that the IVT system is just as effective as conventional therapy in managing pain after TKA.

Pain has been even evaluated with the NRS scale in four studies. Lawford et al. showed that within the intervention group, for each additional unit of baseline pain self-efficacy, pain at 3 months decreased by an additional average of 0.53 (95% CI: 0.28–0.78) NRS units [16]. The study of Moutzouri et al. [17] showed that the minimal clinically important difference for the NPRS was set at 2 points (30%) for chronic pain conditions; the intervention group achieved this target at 6 weeks (2.1-point difference) and at 12 weeks (approximately 3.0-point difference). Gur et al. [20] showed that at baseline, the groups were similar in terms of pain intensity (e.g., pain at rest: VR group 8 (7–9), exercise group 8 (5–9), *p* = 0.694). From pre-treatment to post-treatment patients in the VR group showed a decrease from 8 to 2 in pain at rest, from 9 to 2 in pain during the night and from 9 to 4 in the pain during the time up and go test. Decreases were seen even in the control group with results going from 8 to 5, 9 to 6 and 9 to 6 in the respective previously mentioned groups. The fourth study that considered the NRS [27] using the generalized estimating equation (GEE) model revealed that there was no significant interaction between group and time (*p* = 0.489) for NRS scores. This showed that the reduction in pain did not differ significantly between the two rehabilitation approaches over time. However, both groups showed significant improvement over time (*p* < 0.001) in terms of pain, function, and quality of life. This suggests a reduction in pain in both types of rehabilitation.

Another three studies analyzed pain through the WOMAC subscale for pain. Runkai Zhao et al. [24] showed that six weeks after surgery, patients in the telerehabilitation group experienced less pain than those in the control group. This suggests that the telerehabilitation part of the therapy helped ease pain more quickly, making it a better option than recovering at home without guided support in the early weeks after surgery. Patrick Doiron-Cadrin et al. [25] showed a −0.5 ± 4.5 mean difference in the telerehabilitation group and −1.4 ± 2.4 in the in-person group and the same values were found in the control group showing that there were no benefits in the pre- and post-pain aspect. An et al. [22], in patients undergoing TKA, showed results of the self-reported WOMAC questionnaire, which showed a significant time effect on pain score, significant differences between groups, and a significant interaction between time and group.

Two studies analyzed pain with other scales. Rini et al. [18] with the PAIN coach scales demonstrated that women in the intervention group had significant reductions in pain from baseline to midpoint and post-intervention, while women in the control group did not show a corresponding significant reduction in pain. Bettger et al. [26] showed that both groups experienced a significant reduction in pain scores from baseline to 12 weeks, with no major differences in the overall improvement.

### 3.3. Stability

Among the included studies, four studies evaluated the effect of the use of new technologies on stability aspects. One study [19] showed positive aspects regarding stability, whilst three studies had no significant differences between groups. All the studies taken into consideration have a different modality to evaluate stability making the results difficult to compare.

The study performed by Oliveira et al. [19] used the Mini-BESTest and reported improvements in balance for both the conventional therapy and virtual reality (VR) group, with the VR group showing slightly greater gains [F (1, 38) = 65.65; *p* < 0.0001], and both groups showed higher scores after physical therapy with a large effect size [CT (*p* < 0.001; g2P = 0.22) and VR (*p* < 0.001; g2P = 0.20)]. Results pre-intervention were 20.50 ± 4.83 and post-intervention 25.00 ± 4.18.

In contrast, the study conducted by Bettger et al. [26] assessed the stability indirectly through fall incidence and functional outcomes. They found a slightly higher fall rate in the virtual PT group that might indicate a small trade-off in stability when using remote therapy tools. This could be due to the fact that there is no supervision during home-based digital rehabilitation. The Tousignant et al. [21] study assessed balance with the Berg Balance Scale. Both telerehabilitation and usual care groups showed significant improvements over time. Even though no significant differences were found between groups at T1 and T2. Post-treatment (T2–T3), the usual care group had a greater improvement (by 1.6 points), but this difference was not statistically significant. Lastly, Shim et al. [27] evaluated balance with the Berg Balance Scale with results going from 29.8 ± 11.9 to 49.3 ± 8.5 (*p* < 0.001) at the last evaluation. But no statistical difference between groups has been reported.

### 3.4. Functionality

Thirteen RCTs evaluated the improvement in functionality post rehabilitation. The most common tool to evaluate the improvement of the patients in terms of functionality is the WOMAC Index. The studies even used the KOOS scale, the TUG test, the 30 s CRT, ROM, LEAS, 4 m gait speed and manual muscle strength. We saw that seven articles are statistically significant, demonstrating that new technology rehabilitation protocols have major benefits regarding functionality compared to control groups. Five articles ([16,18,19,26,27]) found no differences between groups and only one [21] shows improvement in favor of the control group. The studies have different assessment tools and rating scales, evaluation timing, follow up and type of population taken into consideration.

Functionality was evaluated in the study of Oliveira et al. [19] and results showed that both groups obtained a statistically significant improvement in the physical capacity after therapy, with a mean difference of 13.97 for the control group and 13.99 for the intervention group. Also, Lawford et al. [16] showed that factors such as age, gender, self-efficacy, pain, education, employment status and body mass index, influenced the intervention and this had effects on physical functions at 3 months. The study conducted by Moutzouri et al. [17] showed a gain in physical function for the intervention group of between 30% for the TUG test (*p* < 0.05) and 32.5% for the 30 s chair rise test (30 s CRT) (vs. 18.3% for the control group) (*p* < 0.05) by the end of follow-up. In addition, statistically significant differences were shown in both groups in the KOOS subscales of ADL, sports and QoL over the 12 weeks. Lastly, statistically significant differences between-groups were found in the Lower Extremity Activity Scale (LEAS), showing greater improvement in the intervention group at 6 weeks. Results were maintained over the 12 weeks of follow-up.

Rini et al. [18] assessed how much pain influenced joint functionality in the enrolled patients. These patients showed very low pain-related interference scores at baseline, so it was difficult to see improvements or changes at follow up.

Gür at al. [20] demonstrated that both groups involved improved their function after treatment; however, the addition of VR to the exercises led to significantly greater improvements in time-based functional tests, such as TUG and SCT, compared to conventional exercises alone. In contrast the results in the WOMAC index were not statistically significant.

Tousignant et al. [21] showed that the control group had greater improvement than the experimental group in functional activities, such as climbing stairs or walking, between T2 and T3 (WOMAC difficulty section, *p* = 0.047). A comparison between T1 and T3 showed that participants from the control group had better physical functioning (*p* = 0.019) and less pain (*p* = 0.013) two months after the end of treatment compared to before treatment. The control group showed better results in some variables at the 2-month follow up compared to the experimental group.

Good results in these types of patients were also reported by An et al. [22], who post-operatively obtained a reduction in the WOMAC index. In the same study, improvements were also seen in the extension strength, in the knee flexion ROM and at the TUG test that decreased, from baseline to 6 weeks post-TKA, by 4.03 s in the PTG group, 2.83 s in the PEG group and 1.36 s in the control group.

Similarly, another study [23] evaluated the WOMAC index and improvements in ROM in patients post-surgery. They found that WOMAC indexes were significantly higher in the experimental group compared to control group (*p* < 0.05), and knee ROM were significantly higher in the experimental group than in the control group with a *p* < 0.05.

The study performed by Bettger et al. [26] found that virtual physical therapy was not inferior to traditional physical therapy in terms of functional status (KOOS) at 6 and 12 weeks, as well as gait speed, knee extension, and knee flexion at 6 weeks.

The study conducted by Ga Yang Shim et al. [27] evaluated functionality using different scales, including the 4 m gait speed as a primary outcome and WOMAC index), ROM, and manual muscle test (MMT) scores as secondary outcomes. Gait speed improved significantly over time in both groups, but there was no significant difference between the groups. WOMAC, BBS, ROM, and muscle tests showed significant improvements over time in both groups, but no statistically significant differences were observed between the two groups. In summary, the study demonstrated that both groups (augmented reality-based digital rehabilitation and conventional rehabilitation) showed significant improvements in all functionality measures over time after total knee arthroplasty. However, no statistically significant differences in functional improvement were found between the two groups for most of the measures analyzed.

In Trevor G Russell et al.’s [28] study, both groups demonstrated significant and clinically meaningful improvements in WOMAC scores, but a significant difference was observed only in the WOMAC stiffness subscale in favor of the tele-rehabilitation group. For the Patient-Specific Functional Scale (PSFS), a significantly greater improvement was observed in the telerehabilitation group compared to the conventional rehabilitation group. In terms of knee ROM (flexion and extension), muscle strength (quadriceps lag) and gait, both groups showed improvements, but no significant differences were found between the groups.

In the study performed by Zhao et al. [24] six weeks after surgery, patients in the telerehabilitation group showed a lower WOMAC score than patients in the control group (*p* = 0.036). Also, the authors evaluated the single-leg stance test (SLST) and the five-times sit-to-stand test (5×SST) showing better results in the telerehabilitation group on both tests (respectively, *p* = 0.026 and *p* = 0.043). Lastly, both groups gained an improvement in joint ROM at 6 and 12 weeks after surgery, which was greater for the telerehabilitation group compared to the control group.

In the study of Piqueras et al. [29], functionality was evaluated and assessed, taking into consideration active knee range of motion (flexion and extension), quadriceps and hamstring muscle strength, TUG test and the functional capacity component of the WOMAC index. The 2-week interactive virtual telerehabilitation (IVT) program has been seen to be as effective as conventional therapy in improving physical function in patients following total knee arthroplasty. Patients achieved functional improvements comparable to those in the conventional therapy group. However, despite this overall equivalence, the study found that the IVT group showed a significantly greater increase in quadriceps strength compared to the control group.

**Figure 2:**
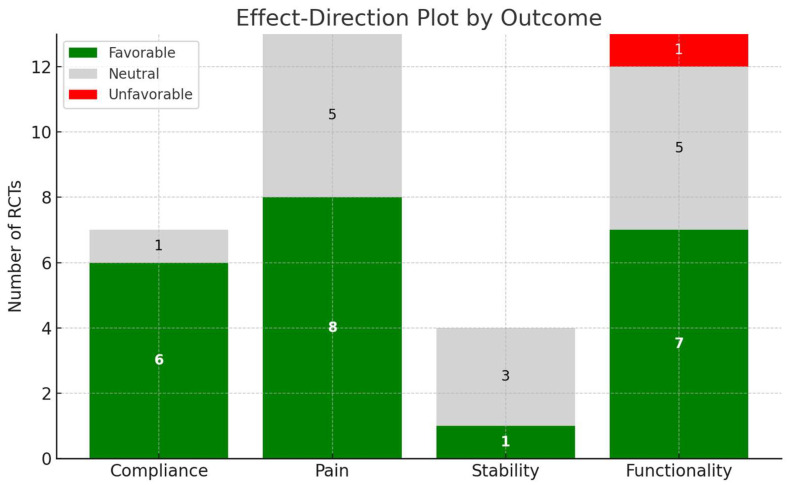
Effect–direction plot of included RCTs by outcome (chart obtained by artificial intelligence).

Results are reported and summarized in an effect–direction plot by outcome (Figure 2).

### 3.5. Risk of Bias

**Figure 3:**
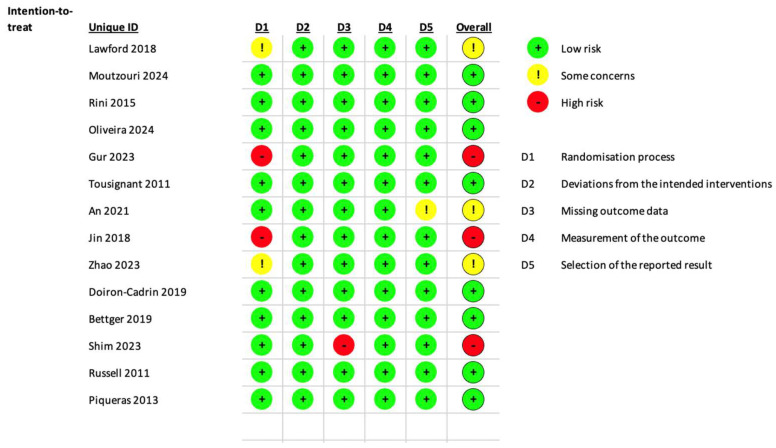
Risk of bias of the studies included in the systematic review [16,17,18,19,20,21,22,23,24,25,26,27,28,29].

The risk of bias in the included studies was assessed using the Risk of Bias 2 (RoB 2) tool. Of the 14 studies included, 8 (60%) were judged to be at low risk of bias, 3 (20%) gave some concerns (i.e., intermediate risk), and the remaining 3 (20%) at high risk of bias. This distribution suggests that the most of the included studies are of adequate methodological quality, although a non-negligible proportion presents limitations that could potentially affect the overall interpretation of the results. The results of the assessed risk of bias are summarized in Figure 3.

## 4. Discussions

This systematic review examined the use of new technologies such as telerehabilitation, virtual reality and exergames in elderly patients diagnosed with knee osteoarthritis, as well as in individuals who underwent total knee arthroplasty due to the condition. Giving patients a positive prospective quality of life, pain relief, and functional improvement is crucial for TKA and KO postoperative management. This is why post-operative rehabilitation encourages recovery and quality of life [30]. Despite the research of the existing literature, the number of studies and systematic reviews specifically addressing this topic remains limited. Notably, there is a clear gap in the literature regarding studies that concurrently include older adults (aged 60 years and above) with osteoarthritis or have undergone total knee arthroplasty. This aspect is due to the fact the new technologies have always been more associated with neurological pathologies ignoring musculoskeletal aspects. Instead, since many psychosocial aspects could influence recovery in these patients, new technologies could be very important [31].

### 4.1. Compliance to the Therapy

Analyzing the results of the seven studies that specifically examined the potential of rehabilitation treatments delivered through new technologies gave us insight regarding compliance to the therapy. Findings suggest that digital health interventions can significantly improve patient adherence to rehabilitation protocols in individuals with knee OA and those recovering from TKA. Platforms that provided structured, interactive, and accessible formats, such as Pain COACH [16] and smartphone-based telerehabilitation [22] were associated with high levels of both compliance and satisfaction.

Furthermore, the possibility to tailor the rehabilitation treatment to individual patient needs and to deliver it remotely, at the patients’ home, seems to promote patient engagement, since this gives patients greater flexibility and autonomy on when to perform the exercises and reduces travel issues that could occur. This is especially relevant for older adults, who may benefit from reduced travel demands and increased comfort in their home setting. This feature represents a potentially time- and cost-efficient alternative, particularly valuable during the immediate postoperative period, when mobility is limited, and patients cannot drive. Telerehabilitation, in general, has been associated with reduced costs compared to in-person therapy. However, the accessibility of telerehabilitation may still be limited by the cost and availability of qualified therapists [32].

However, technical functionality and use remain critical issues for the adherence aspect. Systems that are perceived as difficult to use or to install, may tend to make patients to be less consistent despite the clinical benefits. These findings highlight the importance of developing easy and reliable technologies that reflect the preferences of the target population. Even though there are these difficulties, studies have seen that the treatment could be personalized depending on the abilities and needs of the patient [33].

Nevertheless, the studies analyzed showed that the adherence to these new approaches was consistently higher or equal to the conventional treatments. Further research is needed to examine the long-term clinical outcomes and cost-effectiveness associated with these new technological modalities. In the future, a standardized scale to measure adherence across studies could facilitate comparisons and make studies more significant.

### 4.2. Pain

In our systematic review, we analyzed fifteen studies regarding pain, and of the studies included, we concentrated on the impact of technology-assisted rehabilitation interventions on pain perception. Various technologies were assessed for their ability to reduce pain when compared to conventional rehabilitation approaches. Findings highlight the potential of new rehabilitation technologies to reduce pain in both conservative and postoperative management of knee OA. Many digital programs have been found effective in achieving improvements in pain scores, either as standalone treatments or combined with conventional therapy. The variability in the outcomes across the studies highlight important moderating factors such as patient engagement, baseline pain self-efficacy, and the type of technology used. We observed that programs that offered interactivity and patient feedback, like VR or guided telerehabilitation, reported superior outcomes compared to passive and non-personalized approaches.

Analyzing knee-related virtual reality (VR) interventions, they led to a statistically significant reduction in pain, with low heterogeneity, especially in non-immersive VR applications. These findings support the use of VR in managing knee conditions such as osteoarthritis, patellofemoral pain syndrome, and post-operative recovery, including knee arthroplasty and ACL reconstruction [34]. This aligned with our findings.

Despite some studies reporting no significant differences between-groups, the majority demonstrated comparable or superior reductions in pain in the experimental groups. These results support the importance in the future of integrating technology-based interventions into everyday rehabilitation protocols, especially when in-person access is difficult or when patient engagement is a priority.

Furthermore, in line with what is known, constant exercise reduces pain and improves the health of our joints and guarantees our autonomy, because it reduces those processes of mechanical and inflammatory stress that cause our structures to degenerate more rapidly [35].

Future research should further elucidate the mechanisms by which digital rehabilitation influences pain perception. Aspects such as distraction, neuroplasticity, and behavioral activation could have a crucial role. Another important feature is to identify which patient subgroups are more likely to benefit. In the future, standardized rating scales and outcome measures and long-term follow-ups will be necessary to confirm the durability and generalizability of these findings.

### 4.3. Stability

The results analyzed in the three studies that examined stability and balance have shown contrasting results between them. One study [17] shows that the findings support the hypothesis that immersive and sensorimotor environments can enhance postural control and that digital rehabilitation can be effective in improving balance, even though close attention must be paid to patient safety, particularly in remote settings. Balance disorders are an important issue in the elderly population, and it is an important risk factor for falls [36]. So it is important to analyze this outcome, but at the moment no statistically conclusive results were found in any of the studies.

Studies carried out on Parkinson’s Disease patients or stroke showed that these innovative treatments have significant results regarding gait and balance compared to conventional therapies [37]. These findings have raised important considerations for future research and underlined the importance of establishing safety settings in unsupervised telerehabilitation programs, especially in older adults or early post-operative patients.

### 4.4. Functionality

The studies reviewed show that rehabilitation interventions, whether digital or conventional, result in significant improvements in functional outcomes. The use of various scales, tests and indexes such as the Western Ontario and McMaster Universities Osteoarthritis Index (WOMAC), TUG test, and other functional assessments helped analyze the effectiveness of these interventions. However, taking into consideration this heterogeneity in evaluation methods, it was not possible to compare the final results and reach an actual definitive conclusion.

Previous results indicate that internet-based telerehabilitation leads to functional improvements comparable to those achieved with conventional rehabilitation after total knee arthroplasty [26].

In conclusion, after analyzing the results, we can determine that digital interventions could be very important for the future, but at the moment they are not universally superior to conventional rehabilitation. In some analyzed elements, traditional rehabilitation methods still turn out to be more effective. Future studies should focus on refining these digital rehabilitation approaches to enhance their efficacy, particularly in terms of long-term functional outcomes.

Therefore, after carefully examining all the outcomes included in this review, we can expand our discussion by highlighting how these findings contribute to a broader understanding of the effectiveness and applicability of VR-based rehabilitation in clinical practice.

Our findings align with those of Bravi et al., that took into consideration 11 randomized controlled trials and found no significant differences in pain, function, or mobility outcomes between supervised and unsupervised rehabilitation following TKA, suggesting that unsupervised approaches can be a viable and cost-effective alternative to traditional supervised programs [38]. This is an important aspect that relates to and reinforces our systematic review, since new technologies are all based on unsupervised or remote supervision rehabilitation protocols.

In 2019, a review analyzed the efficacy of virtual reality and exergames in the rehabilitation protocols of patients with musculoskeletal system disorders and the results showed positive results and significant results in the outcomes taken into consideration, such as functionality, pain relief, increased range of motion in the joints and reduced symptoms. At that time, no significant efficacy had been demonstrated in non-chronic pathologies. But in recent years, new randomized clinical trials have been carried out, showing the importance of virtual reality and exergames even in other pathologies [39].

Therefore, after carefully examining all the outcomes included in this review, we can expand our discussion by highlighting how these findings contribute to a broader understanding of the effectiveness and applicability of new technology rehabilitation protocols that could be used in clinical practice. Even in hospital settings, they could help to make the patient imagine themself in a different location and reality [40].

These new studies and research show the importance of these new technologies, with keen regard to VR-rehabilitation, showing the importance it will have in the future in many medical settings [41].

## 5. Limitations

This systematic review has some limitations to acknowledge. First, the studies included used a wide range of assessment tools and rating scales; due to [13] this heterogeneity, a consistent, unified analysis across all studies was not feasible. Secondly, a complete comparison between the studies was not possible, due to different outcomes and different technologies used. Thirdly, the studies often took into consideration a small sample size of patients and the variability in the mean age of the participants may have influenced sample homogeneity. Fourthly, the outcomes have not been analyzed and followed in the long-term, so, especially for stability and functionality, no long-term outcomes have been analyzed. In the future, multi-center studies could be helpful in having a major overview and long-term results.

## 6. Conclusions

This systematic review, uniquely focused on patients aged 60 years and older with knee OA or in post-TKA rehabilitation, shows that structured telerehabilitation, non-immersive VR, and interactive platforms with personalized feedback can improve adherence, reduce pain, and enhance functional recovery, often matching or exceeding the results of conventional rehabilitation. Programs such as Pain COACH, Kinect-based VR training, and remote-supervised systems (e.g., VERA, REACTS LiteVR) emerged as the most promising in terms of usability, clinical benefit, and patient satisfaction. Their ability to deliver tailored rehabilitation at home, reduce travel demands, and ensure continuity of care makes them valuable tools, particularly where in-person therapy is not readily accessible. Clinically, these interventions could be integrated into standard rehabilitation pathways to expand access, support long-term engagement, and reduce costs without compromising outcomes. However, stability and fall-risk prevention remain areas requiring further high-quality trials with standardized measures and long-term follow-up. Future research should optimize digital protocols, enhance patient engagement, and tailor interventions to individual profiles, thereby maximizing safety, effectiveness, and sustained benefit.

## Figures and Tables

**Table 1:** Summary of the studies.

Study	Participants	Technologies	Questionnaires, Assessment Tools and Timing	Rehab Protocol	Control Protocol
Belinda J Lawford et al., 2018 [16]	Knee OA Intervention group (*n* = 74), mean age: 60.8 ± 6.5 Control group (*n* = 74), mean age: 61.5 ± 7.6	Educational material + online interactive automated PCST program + physical therapist consultations via Skype	Arthritis Self-Efficacy Scale Numerical Rating Scale (NRS) Western Ontario and McMaster Universities Arthritis Index (WOMAC) Pain Catastrophizing Scale Five-point Likert scale At baseline, 3 months and 9 months	Educational material + an online interactive automated PCST program, involving completion of one 35 to 45 min training module per week for 8 weeks + seven physical therapist consultations via Skype over 12 weeks, with each consultation lasting 30 to 45 min.	Same educational material as the intervention group but did not have access to the PCST program
Maria Moutzouri et al., 2024 [17]	Knee OA Intervention group (*n* = 22), mean age: 65.1 ± 5.3 Control group (*n* = 22), mean age: 63.5 ± 5.6	minA web-based structured video exercise program + KOA disease consultatory video sessions (ESCAPE-pain program) + outdoor PA (physical activity) walk journey	Knee Injury Osteoarthritis Outcome Score (KOOS) Modified Baecke Physical Activity Questionnaire (mBQ) Lower Extremity Activity Scale (LEAS) Tampa Scale of Kinesiophobia (TSK) Numerical Pain Rating Scale (NPRS) 30 s sit-to-stand test Time up-and-go [TUG] test Short-Form 12 (SF-12) Baseline and at 6 weeks	A web-based structured video exercise program (twice a week) + KOA disease consultatory video sessions (once a week) + outdoor PA walk journey 3 times/week for 6 weeks.	General web-based information and advice for KOA and patients were encouraged to follow outdoor PA 5 times/week.
**Study**	**Participants**	**Technologies**	**Questionnaires, Assessment Tools and Timing**	**Rehab Protocol**	**Control Protocol**
Christine Rini et al. [18]	Knee or hip OA Intervention group (*n* = 58), mean age: 68.52 ± 7.65 Control group (*n* = 55), mean age: 66.67 ± 11.02	Pain COACH program	Arthritis Impact Measurement Scale 2 (AIMS2) Arthritis Self-Efficacy Scale (ASES) Pain Anxiety Symptoms Scale (PASS-20) Positive and Negative Affect Scale (PANAS) At baseline, midpoint assessment and post-intervention (9 to 11 weeks after randomization)	Participants in this condition used the Pain COACH program, which translated an in-person pain-coping-skills training (PCST) protocol for delivery via the internet. Participants were led through the program by a “virtual coach”. Eight modules, each one took 35–45 min. One per week.	No access to Pain COACH at any point during or after the study.
Luana Karine Resende Oliveira et al., 2024 [19]	Knee OA Intervention group (*n* = 20), mean age: 62.35 ± 7.39 Control group (*n* = 20), mean age: 62.60 ± 8.62	Virtual reality training using the Xbox 360 videogame with Microsoft Kinect 360 sensor. Game chosen: ‘‘Kinect Adventures’’ with three subgame modes: Reflex Ridge, 20,000 leaks, and RallyBall	Anticipatory postural adjustments (APAs) Mini-Balance Evaluation Systems Test (Mini-BESTest) WOMAC Visual Analog Pain Scale (VAS) at baseline and post-intervention	30 min of kinesiotherapy + 20 min of exercise with non-immersive VR 8-week treatment, 50 min sessions, twice a week	30 min of kinesiotherapy + 20 min of exercise with the objective of training postural control and balance 8-week treatment, 50 min session, twice a week
**Study**	**Participants**	**Technologies**	**Questionnaires, Assessment Tools and Timing**	**Rehab Protocol**	**Control Protocol**
Ozan Gür et al., 2023 [20]	TKA due to osteoarthritis Intervention group (*n* = 10), mean age: 63 Control group (*n* = 11), mean age: 61	BOBOVR Z5 model VR devices	Numerical Pain Rating Scale (NPRS) Standardized Mini-Mental Test Tampa Kinesiophobia Scale Pain Catastrophizing Scale ROM (universal goniometer) Timed up and go test The stair-climb test WOMAC Short form 36 (SF-36) pre- and post-intervention	Exercise program 10 repetitions of each exercise twice a day + immersive VR application in addition to the exercise (10 min a day in the sitting position for 3 weeks, twice a week)	Only exercise program, 10 repetitions of each exercise twice a day
Michel Tousignant et al., 2011 [21]	TKA Experimental group (*n* = 21) Mean age: 66 ± 10 Control (*n* = 20) Mean age 66 ± 13	Videoconferencing CODECs (550 MXP, Tandberg) with remote-controlled cameras, 50 cm LCD screens and associated software for user-friendly control	30 s chair stand ROM (universal goniometer) WOMAC Timed up and go test Tinetti test Berg Balance scale Functional Autonomy Measurement System (SMAF) SF-36 At the beginning (T1) and at the end (T2) of the experimental intervention, and four months afterwards (T3).	The tele-treatments were delivered at a rate of two sessions per week for eight weeks, for a total of 16 sessions.	Conventional rehabilitation
**Study**	**Participants**	**Technologies**	**Questionnaires, Assessment Tools and Timing**	**Rehab Protocol**	**Control Protocol**
Jung Ae An et al., 2021 [22]	Candidates for TKA with advanced knee OA Preoperative telerehabilitation group—PTG (*n* = 18), mean age: 71.1 ± 3.30 Preoperative patient education group—PEG (*n* = 17), mean age: 70.05 ± 2.41 Control (*n* = 18), mean age: 70.38 ± 2.59	Telerehabilitation at home using a smartphone or tablet via a two-way video call	Isokinetic Strength Assessment WOMAC Knee Flexion-ROM Timed up and go (TUG) Test Pressure pain threshold (PPT) At 4 weeks preoperatively, post-interventionally, and 6 weeks after TKA	The PTG group took part in a 30 min/session, 2 times/day, 5 days/week for 3 weeks, for a total of 30 sessions before TKA. The PEG group participated in a preoperative education session, a non-supervised intervention performed for 30 min per session, 2 times/day, 5 days/week for 3 weeks, and the exercise timing was adjustable.	The control group received the usual care, such as following the guideline of surgical procedure, postoperative progress monitoring, discharge destination determination, and simple quadriceps exercise intervention.
Chi Jin et al., 2018 [23]	TKA in patients with knee OA Experimental (*n* = 33); mean age: 66.45 ± 3.49 Control (*n* = 33); mean age: 66.30 ± 4.41	Games in an immersive virtual environment. Mide Technology Inc., Cangzhou, China.	WOMAC Hospital for Special Surgery Knee Score (HSS) Visual Analog Scale (VAS) Range of motion (ROM) Before TKA, 1 month after, 3 months after and 6 months after TKA	Exercises + VR intervention was applied in the experimental group beginning the second day after TKA. Patients were asked to row a boat using knee flexion in an immersive virtual environment for 30 min periods, three times a day.	Active and passive exercises. Patients in the control group were asked to flex their knees passively using their arms until pain tolerance was reached.
**Study**	**Participants**	**Technologies**	**Questionnaires, Assessment Tools and Timing**	**Rehab Protocol**	**Control Protocol**
Runkai Zhao et al., 2023 [24]	TKA in patients with knee osteoarthritis Experimental group (*n* = 50), mean age: 65 Control group (*n* = 50), mean age: 65	The Vital Health Remote Rehabilitation System: patient-side APP, wearable sensors, and surgeon-side websites	Knee range of motion (ROM) WOMAC Knee Society Score (KSS) SF-36 Five times sit-to-stand Test Single-leg stance Test (SLST) At 2, 6, and 12 weeks after surgery	Postoperative rehabilitation protocol on the APP for 3 months + evaluation of the performance in terms of the quality and quantity of training movements completed every 2 weeks and generated a rehabilitation report and a new training plan for the next two weeks	Patients in the control group underwent home-based rehabilitation following written instructions, with regular outpatient clinic visits with a follow-up of 3 month
Patrick Doiron-Cadrin et al., 2018 [25]	TKA candidates with severe knee OA Telerehabilitation (*n* = 12) Mean age: 69.9 ± 9.1 In person (*n* = 11) Mean age: 61.3 ± 8.1 Control (*n* = 11) Mean age: 66.7 ± 9.2	Telerehabilitation with REACTS LiteVR medical consultation application	Lower Extremity Functional Scale (LEFS) WOMAC The Short Form (36) Health Survey (SF-36) Global Rating of Change scale (GRC) Timed up and go (TUG) Self-paced walk (SPW) Timed stair tests (ST) Satisfaction questionnaire At baseline and after 12 weeks	The in-person prehabilitation group received a 12-week rehabilitation program. Tele-prehabilitation group received the same exercise program and advice through an internet-based telecommunication mobile application after initial assessment.	Usual care
**Study**	**Participants**	**Technologies**	**Questionnaires, Assessment Tools and Timing**	**Rehab Protocol**	**Control Protocol**
Prvu Bettger et al., 2020 [26]	TKA for non-traumatic causes Virtual physical therapy (PT) Group (*n* = 153) Mean age: 65.4 ± 7.7 Traditional physical therapy (PT) Group (*n* = 153) Mean age: 65.1 ± 9.2	VERA system (Virtual Exercise Rehabilitation Assistant)	Knee Injury and Osteoarthritis Outcome Score—KOOS Patient-Reported Outcomes Measurement Information System (PROMIS) 10-item global health assessment Patient-Specific Functional Scale Physical activity 10 m walk test ROM knee At baseline, at 6 weeks post-discharge and at 12 weeks post-discharge	Post-surgery patients used the system immediately after hospital discharge to view their own progress and could perform exercises as often as desired, with no restrictions on usage frequency or duration. Patients in the virtual PT group engaged for 5.9 ± 1.7 days per week.	Patients received usual physical therapy sessions at their residence or could visit clinics for their therapy sessions. Patients typically engaged in physical therapy 3.3 ± 2.0 days per week on average.
Ga Yang Shim et al., 2023 [27]	TKA Digital healthcare rehabilitation (DR) group (*n* = 28) Mean age: 68.25 ± 5.80 Conventional rehabilitation (CR) group (*n* = 28) Mean age: 72.96 ± 4.56	Brochure-based exercises + AR-based digital healthcare system (UINCARE Home +; UINCARE Corp., Seoul, Republic of Korea)	4 m gait speed WOMAC Health-related quality of life (EQ5D5L) Pain NRS Berg Balance Scale (BBS) Range of motion (ROM) Satisfaction questionnaire Manual muscle yest (MMT) At baseline (T0) and 3 (T1), 12 (T2), and 24 (T3) weeks after randomization.	Brochure-based exercises for 3 weeks followed by AR-based exercises for 9 weeks, 30 min per session at 12 levels of intensity. Each session consisted of 10 motions, 3 sets of 10 repetitions.	The control group performed only brochure-based exercises for 12 weeks, according to the standard rehabilitation protocol for patients after TKA
**Study**	**Participants**	**Technologies**	**Questionnaires, Assessment Tools and Timing**	**Rehab Protocol**	**Control Protocol**
Trevor G Russell et al., 2011 [28]	TKA Telerehabilitation group (*n* = 34) Control group (*n* = 31) Mean age: 68 ± 7.9 years	A computer-based telerehabilitation system specifically engineered for this study	WOMAC The Patient-Specific Functional Scale The Spitzer Quality-of-Life Uniscale Timed up and go test Pain intensity (VAS) Knee ROM Quadriceps muscle strength Gait Assessment Rating Scale Limb girth measurements Baseline and after 6 weeks from TKA	Forty-five minutes sessions, during which physical therapists administered, a rehabilitation program that consisted of self-applied techniques under the guidance of the remote therapist, along with exercises and education in the postoperative management of the total knee replacement.	Rehabilitation was administered in an outpatient physical therapy department, according to standard clinical protocol. Intervention sessions were of forty-five minutes.
Mercè Piqueras et al., 2013 [29]	TKA for knee OA Control group (*n* = 70) Intervention group (*n* = 72) Mean age: 73.3 ± 6.5	Interactive virtual telerehabilitation (IVT)	ROM (goniometer) Quadriceps strength dynamometer Nicholas manual muscle tester (NMMT) Hamstring strength VAS Timed up and go test WOMAC Baseline, after completing rehabilitation and at 3-month follow-up	The group IVT received 1 h sessions for 10 days	The control group received the standard clinical protocol of TKA rehabilitation consisting of 1 h sessions for 10 days

## Data Availability

The data that support the findings of this study are available from the corresponding author upon reasonable request.
